# Addressing Meta-Inflammation in the Comprehensive Management of Chronic Pain

**DOI:** 10.7759/cureus.94863

**Published:** 2025-10-18

**Authors:** Morgan McMasters, Jorge Mora

**Affiliations:** 1 Anesthesiology and Critical Care, Florida International University, Herbert Wertheim College of Medicine, Miami, USA; 2 Translational Medicine, Florida International University, Herbert Wertheim College of Medicine, Miami, USA

**Keywords:** chronic pain disorders, chronic pain management, metabolic inflammation, meta-inflammation, neuroinflammation

## Abstract

Chronic pain is a widespread condition affecting millions globally, placing significant strain on healthcare systems, as evidenced by the current opioid crisis. Despite advances in pain management, conventional treatments often fail to address the underlying inflammatory mechanisms that sustain chronic pain. Recent evidence suggests that metaflammation, or meta-inflammation - defined as a chronic, low-grade inflammatory state driven by metabolic dysregulation - plays a pivotal role in the persistence and exacerbation of chronic pain. This review explores the relationship between meta-inflammation and chronic pain, emphasizing the contribution of inflammatory mediators such as tumor necrosis factor-alpha (TNF-α), interleukin (IL)-6, and IL-1β.

Meta-inflammation is induced by metabolic imbalances, poor dietary habits, gut microbiota alterations, and other lifestyle factors, leading to systemic immune activation. Persistent cytokine signaling results in neuroinflammation, promoting central sensitization and heightened pain perception. Chronic diseases such as obesity, diabetes, cardiovascular disease, and endometriosis share common inflammatory pathways with chronic pain, further supporting the role of meta-inflammation in pain pathophysiology.

Given the association between metabolic dysfunction and chronic pain, targeting meta-inflammation presents a novel therapeutic approach to pain management. Dietary modifications, such as the Mediterranean and DASH (Dietary Approaches to Stop Hypertension) diets, supplementation with omega-3 fatty acids, polyphenols, curcumin, and vitamin D, have demonstrated anti-inflammatory and pain-modulating effects. Additionally, lifestyle interventions, such as optimizing sleep quality, and medical management for metabolic dysfunction - such as metformin, glucagon-like peptide-1 (GLP-1) agonists, and sodium-glucose cotransporter-2 (SGLT-2) inhibitors - offer promising avenues for reducing systemic inflammation and chronic pain symptoms.

This review underscores the necessity of integrating meta-inflammation management into standard pain treatment protocols. By incorporating targeted dietary, lifestyle, and pharmacologic strategies, clinicians can more effectively address the underlying inflammatory drivers of chronic pain, ultimately improving patient outcomes and reducing reliance on opioids.

## Introduction and background

Background

Chronic pain places a significant strain on healthcare systems globally. In the United States, the CDC estimates that 25.4% of adults experience chronic pain [1]. Managing chronic pain has become a central issue, especially considering the ongoing opioid epidemic. Since the introduction of opioids in the 1990s, opioid-related drug overdose deaths have risen steadily [2]. Between 1999 and 2017, deaths from prescription opioid overdoses in the U.S. increased from 3,442 to 17,029 people [2]. Pain management has since evolved into an entire subspecialty of medicine, with pain management specialists addressing chronic pain symptoms in daily practice.

The molecular basis of pain involves the activation of nociceptors in the peripheral nervous system by inflammatory molecules. Nociceptors respond to noxious or sub-noxious stimuli, such as prostaglandins and bradykinins, which trigger calcium channel opening and subsequent cellular depolarization [3]. This depolarization leads to the release of pro-inflammatory cytokines, which activate immune cells, smooth muscle cells, and the surrounding tissue [3]. The resulting inflammation not only triggers pain but also propagates and reinforces pain sensation through the continued activation of nociceptors.

Prolonged inflammatory states create an environment rich in pro-inflammatory molecules, such as tumor necrosis factor-alpha (TNF-α), interleukin (IL)-6, IL-1β, nerve growth factor (NGF), prostaglandin E2 (PGE2), chemokine (C-C motif) ligand 2 (CCL2), and chemokine (C-X-C motif) ligand 1 (CXCL1) [4]. This chronic exposure can lead to allodynia - excessive pain in response to stimuli that typically do not cause pain - by increasing both the number and sensitivity of nociceptors and immune cells [3,4]. Chronic inflammatory states, often associated with aging, diabetes, and meta-inflammation, play a key role in maintaining pain signaling over time.

Indications for management 

The International Association for the Study of Pain defines pain as “an unpleasant sensory and emotional experience associated with, or resembling that associated with, actual or potential tissue damage,” and chronic pain develops when pain persists beyond an expected 6-12 weeks following injury [5]. Patients may be referred to specialty pain clinic care as an adjunct to other pain management options - such as pain not responsive to initial conservative care, or a desire to avoid surgery.

Additionally, referral to a pain clinic may be made for patients with complex, difficult-to-treat pain: persistent pain that significantly impacts function, quality of life, anxiety and/or depression; persistent pain that cannot be explained and requires treatment; pain requiring interventional treatment; high-risk or complex pain-related pharmacology or polypharmacy; persistent neuropathic pain that has not responded to first-line treatments; and uncertainty regarding diagnosis or treatment for resistant widespread pain.

Once referral to a pain clinic occurs, the guiding principle is that these clinics provide comprehensive care, including a biopsychosocial approach that identifies all aspects contributing to a patient's diagnosis and symptoms [6]. A systematic review on the benefits of comprehensive pain management approaches found moderate improvement in overall function, as well as in short- and long-term pain outcomes, in patients receiving early multidisciplinary and holistic care [7]. It can therefore be inferred that incorporating meta-inflammation into the pain management plan from the first visit would not only be more comprehensive, but also provide the best outcomes for these patients.

Current pain management approach

The American Society of Anesthesiologists defines chronic pain as "pain of any etiology not directly related to neoplastic involvement, associated with a chronic medical condition, or extending in duration beyond the expected temporal boundary of tissue injury and normal healing, adversely affecting the individual's function or well-being" [8]. The goal of their guidelines is to effectively reduce pain, improve function, and minimize psychosocial suffering. A multimodal, long-term approach is emphasized for comprehensive pain management [8].

The guidelines recommend 12 treatment modalities, including pharmacologic management, physical therapy, psychological therapy, and others (summarized in Table 1).

**Table 1:** Summary of pain current management modalities as defined by the American Society of Anesthesiology Table credit: Rosenquist et al. [8]

Treatment Modality	Description
Ablative	Chemical denervation, cryoneurolysis or cryoablation, thermal intradiscal procedures (i.e., intervertebral disc annuloplasty (IDET), transdiscal biacuplasty), and radiofrequency ablation
Acupuncture	Traditional acupuncture, electrical acupuncture
Blocks	Joint blocks and nerve or nerve root blocks
Botulinum toxin injections	-
Electric nerve stimulation	Neuromodulation with electrical stimulus (i.e., subcutaneous peripheral nerve stimulation and spinal cord stimulation) and transcutaneous electrical nerve stimulation (TENS)
Epidural steroid injections	-
Intrathecal drug injections	Intrathecal neurolytic blocks, intrathecal nonopioid injections (e.g., steroids, ziconotide, local anesthetics), and intrathecal opioid injections
Minimally invasive spinal procedures	Vertebroplasty, kyphoplasty, and percutaneous disc decompression (e.g., nucleoplasty or coblation)
Pharmacologic management	Anticonvulsants, antidepressants, benzodiazepines, N-methyl-D-aspartate (NMDA) receptor antagonists, nonsteroidal anti-inflammatory drugs (NSAIDs), opioid therapy (e.g., oral, transdermal, transmucosal, intranasal, and sublingual), skeletal muscle relaxants, and topical agents (e.g., lidocaine, capsaicin, and ketamine)
Physical therapy	Physiotherapy, fitness classes, and exercise therapy
Psychological therapy	Cognitive behavioral therapy, biofeedback, relaxation training, supportive psychotherapy, group therapy, or counseling
Trigger point injections	Direct injections, especially for myofascial pain

Despite the knowledge that all pain stems from inflammatory mediation, addressing systemic inflammation, specifically meta-inflammation, is notably absent from these strategies. This review aims to define meta-inflammation, explore its role in chronic pain, and propose addressing it as part of a comprehensive approach to pain management.

Meta-inflammation

Meta-inflammation refers to a low-grade, chronic inflammatory process mediated by macrophages and induced by metabolic imbalances, particularly in the context of high-fat, high-fructose, and calorie-dense diets [9,10]. These macrophages can be activated in several tissues, including the colon, muscle, and adipose tissue [11]. Poor diet, aging, changes in gut microbiota [2], lifestyle, and environmental factors lead to the activation of macrophages, which secrete cytokines that recruit additional immune cells [12]. This process is compounded by a reduction in adipose tissue regulatory B cells, which normally produce anti-inflammatory molecules such as IL-10 and transforming growth factor beta-1 (TGF-β1) [12].

Further, caspase-1 and inflammasome activation play critical roles in adipocyte function and metabolism. Studies in mice show that a deficiency in caspase-1 or the inflammasome component NLRP3 (NOD-, LRR-, and pyrin domain-containing protein 3) leads to improved insulin sensitivity and mitochondrial function, suggesting a potential target for novel therapies aimed at reducing metabolic inflammation [13].

Overall, the metabolic disturbance results in an increased presence of circulating and resident adipose tissue monocytes, increased circulating cytokines, and decreased activation of regulatory lymphocytes. All culminate to produce a chronic, low-grade, and maladaptive inflammatory state.

**Figure 1:**
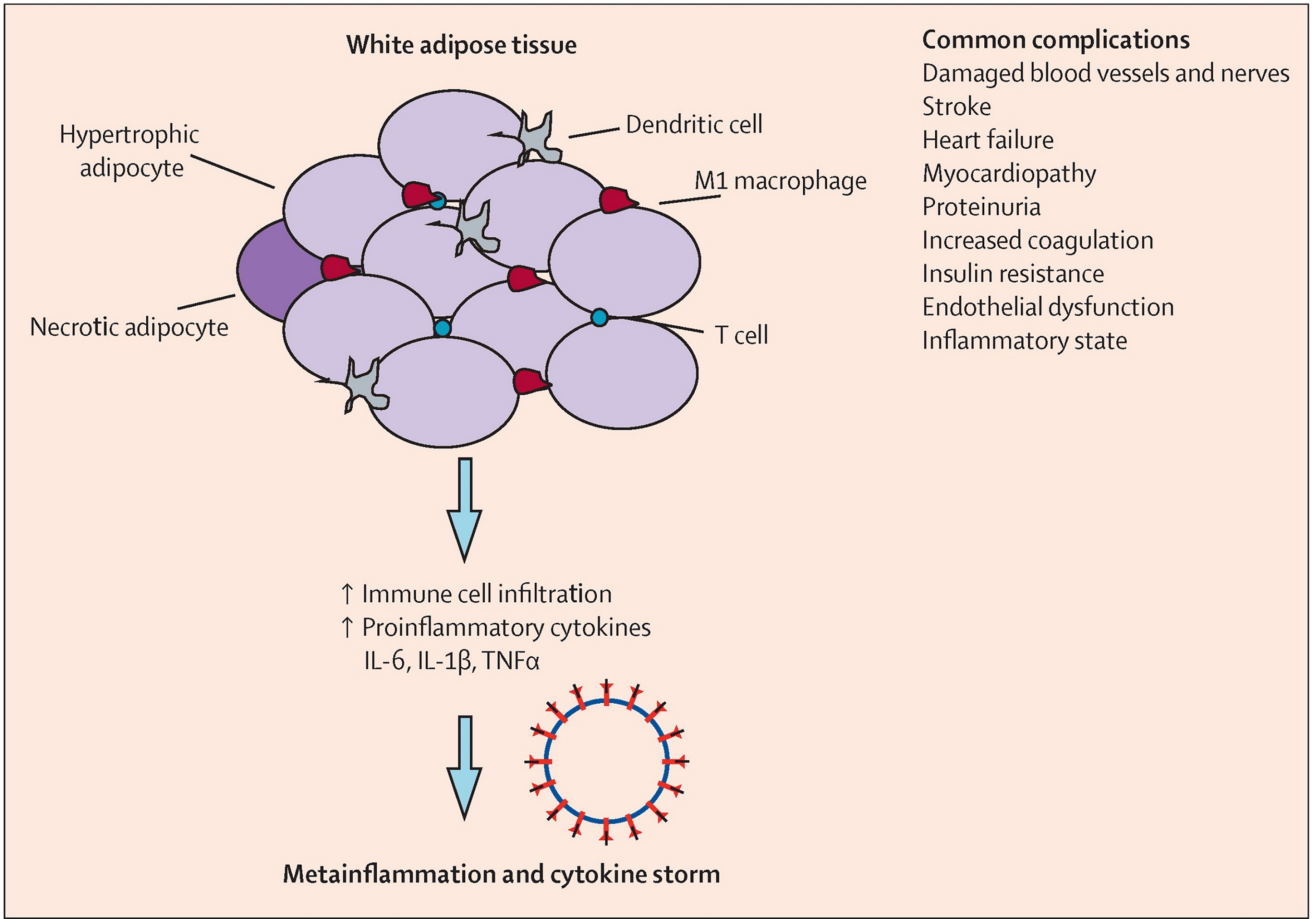
Immune activation and inflammatory signaling in white adipose tissue contribute to meta-inflammation White adipose tissue in metabolic disease becomes inflamed due to the accumulation of hypertrophic and necrotic adipocytes, which recruit immune cells including dendritic cells, M1 macrophages, and T cells. These immune cells secrete proinflammatory cytokines such as interleukin (IL)-6, IL-1β, and tumor necrosis factor-alpha (TNF-α), promoting further immune infiltration and sustaining chronic inflammation. This low-grade, persistent inflammation, termed meta-inflammation, may escalate into a cytokine storm and contribute to complications such as vascular damage, heart failure, insulin resistance, and endothelial dysfunction. Image credit: Reprinted from Steenblock et al. [14]

One proposed mechanism of meta-inflammation from adipose tissue is illustrated in Figure 1.

In the normal state, resident adipose tissue macrophages (ATMs) mostly show an M2-like phenotype, and regulatory T cells and eosinophils support these ATMs to maintain an anti-inflammatory state [11]. In the presence of excessive caloric, fat, or fructose intake, there is increased adipocyte chemokine production, which recruits monocytes from circulation and promotes the proliferation of ATMs [11]. Most of the activated monocytes and ATMs express CD11c and/or CD9 and the M1-like polarized phenotype. A decreased number of eosinophils and regulatory T cells (Tregs), along with an increased number of neutrophils, type 1 innate lymphoid cells (ILC1), CD8+ T cells, T helper type 1 cells (Th1), and B2 cells, enhance M1-like ATM polarization, producing excess adipose tissue inflammation [11].

## Review

Methods

We conducted a comprehensive literature search using PubMed to identify studies examining the relationship between metabolic inflammation (meta-inflammation), neuroinflammation, and chronic pain conditions. Searches were performed iteratively between January and September 2025 and used a combination of Medical Subject Headings (MeSH) terms and free-text keywords. Representative search strings included: "metabolic inflammation" OR "meta-inflammation" AND "chronic pain", "neuroinflammation" AND "glial activation" AND "hyperalgesia", "systemic inflammation" AND ("migraine", "chronic low back pain", OR "widespread pain"), "cytokines" OR "chemokines" AND "central sensitization", "diet" OR "dietary interventions" OR "Mediterranean diet" AND "inflammation" AND "chronic pain", "microbiome" OR "short-chain fatty acids" AND "pain", and "sleep deprivation" OR "sleep disturbance" AND "CRP" OR "interleukin-6".

Searches were refined as follows: initial results were reviewed for relevance, and additional keywords were developed based on recurring mechanistic pathways and risk factors (e.g., adipose tissue inflammation, immune-metabolic signaling, and lifestyle interventions). We also reviewed the reference lists of included studies and used PubMed’s “similar articles” feature to capture additional publications.

Priority was given to population-based epidemiologic studies and hypothesis-driven research, as well as systematic reviews published in peer-reviewed journals within the last 10-15 years, although older studies were included when foundational to the field (e.g., early descriptions of glial activation in central sensitization). Studies were screened for relevance to the review objectives, focusing on mechanistic links between metabolic dysregulation, systemic inflammatory mediators (e.g., C-reactive protein (CRP), IL-6, and TNF), and pain sensitization.

The exclusion criteria included (i) articles not available in English; (ii) single case reports; and (iii) studies lacking mechanistic, epidemiologic, or clinically relevant data.

This multi-step, iterative approach allowed us to integrate a broad but targeted set of evidence, capturing both classical neuroinflammation studies and newer literature linking meta-inflammation, diet, microbiome, sleep, and systemic immune activation to chronic pain outcomes.

Literature results

A PubMed keyword search on "metabolic inflammation AND pain" yielded numerous results demonstrating how pain syndromes may be associated with a metabolic response that mediates a pro-inflammatory state. Meta-inflammation, defined as chronic, low-grade inflammation arising from metabolic dysregulation (e.g., obesity and insulin resistance), should be distinguished from general neuroinflammation, as it links peripheral immune activation with systemic comorbidities and chronic disease risk. Both localized and widespread pain have been associated with neuroinflammation driven by glial activation, with central cytokines and chemokines acting as potent neuromodulators to induce hyperalgesia and allodynia following central nervous system (CNS) exposure [15]. Sustained elevations in cytokines and chemokines, as occur in metabolic imbalance, sensitize local nociceptors and CNS glia, increasing vulnerability to chronic pain via central sensitization. Therefore, neuroinflammation drives widespread chronic pain via central sensitization. While neuroinflammation refers specifically to this central sensitization, meta-inflammation refers to the broader impact of sustained increases in inflammatory mediators on all the metabolic processes of the body. 

Recent population-based studies reinforce this model by demonstrating that systemic inflammation attenuates the protective effects of physical activity on chronic pain outcomes. It was shown that vigorous physical activity was protective against chronic neck, back, and hip pain, as well as migraine, but this association was significantly blunted among individuals with elevated BMI and high CRP, underscoring the modifying role of systemic inflammation on pain pathways [16]. Similarly, Jia et al. (2025) found that inflammatory markers such as CRP, white blood cell levels, and particularly neutrophils partially mediated the normally inverse relationship between muscle mass and migraine risk, suggesting a mechanistic link between systemic inflammatory load and pain susceptibility [17]. Mendelian randomization studies have further clarified causal relationships between inflammatory regulators (e.g., interferon gamma (IFN-γ), monocyte chemoattractant protein-3 (MCP-3), and IL-16) and neuropathic pain phenotypes, supporting inflammation as a key driver in neuropathic pain [18].

Collectively, these findings indicate that chronic pain emerges from an interplay between localized neuroinflammatory mechanisms and systemic metabolic dysregulation, with meta-inflammation acting as a bridge. This highlights the importance of considering comorbidities such as obesity, metabolic syndrome, and systemic inflammatory burden when designing pain management strategies. Emerging evidence also suggests that targeting diet, sleep, microbiome composition, and promoting vigorous physical activity may attenuate systemic inflammation, restore immune homeostasis, and enhance pain resilience, leading to promising directions for personalized interventions.

Meta-inflammation associated with chronic pain and chronic disease

**Figure 2:**
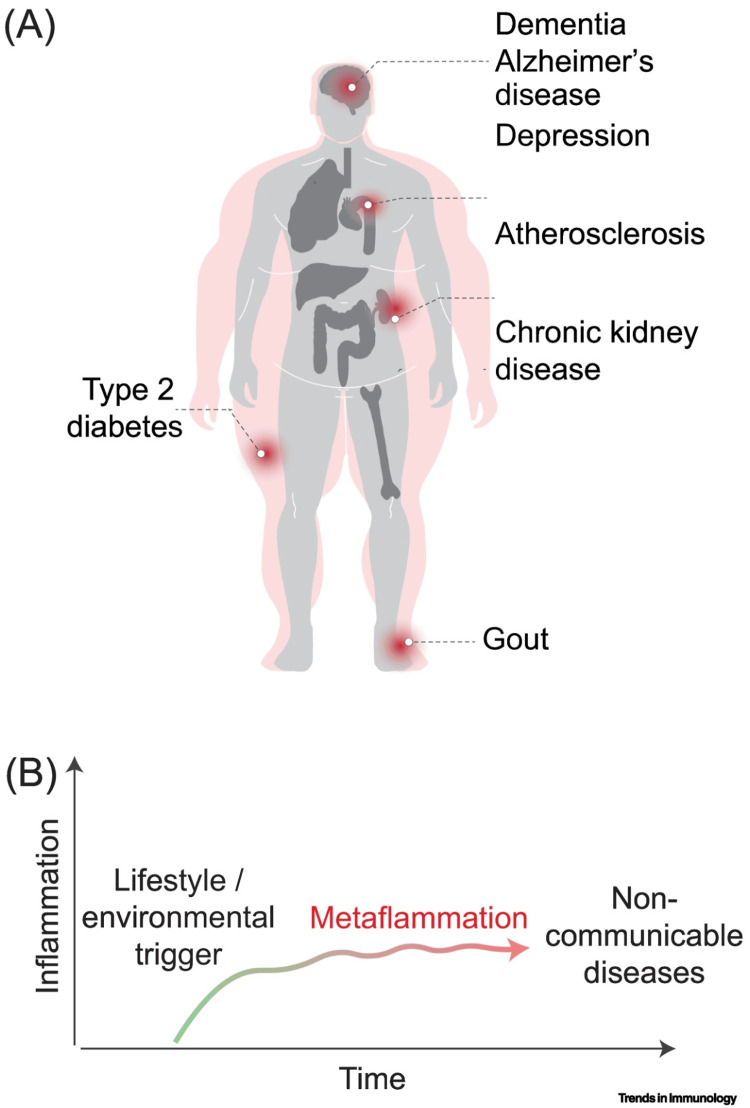
Progression of meta-inflammation and its links to chronic disease (A) Meta-inflammation is implicated in the pathogenesis of multiple non-communicable diseases, including dementia, Alzheimer’s disease, depression, atherosclerosis, chronic kidney disease, type 2 diabetes, and gout. (B) Following lifestyle or environmental triggers (such as diet, stress, or physical inactivity), inflammation gradually increases and becomes persistent, leading to "metaflammation" or meta-inflammation. Over time, this sustained inflammatory state contributes to the development of chronic diseases. Image credit: Reprinted from Picon-Galindo et al. [23]

Meta-inflammation contributes to a variety of chronic diseases, including obesity, diabetes, and cardiovascular disease, which are often comorbid with chronic pain [10]. For example, in chronic kidney disease (CKD) and congestive heart failure (CHF), increased inflammatory markers are closely linked to disease progression [19]. As discussed previously, inflammatory mediators such as histamine, bradykinins, interleukins, tumor necrosis factor, and leukotrienes contribute to both inflammation and pain. These inflammatory markers are elevated as a result of certain diets, fatty acid imbalances, sleep deprivation, hyperglycemia, or saturated fats, among others, promoting “inflammatory states, direct organ damage, and pain” [20,21]. Endometriosis, complicated by chronic pelvic pain, has also been shown to be mediated by neuroinflammation and microbial dysbiosis. New research on managing chronic pelvic pain is aimed at healing the microbiome and reducing chronic, low-level inflammatory states through dietary interventions and nutritional supplements targeting inflammation, oxidative stress, and the microbiome [22]. Figure 2 illustrates the relationship between the inflammatory state and chronic disease.

Microbiome alteration

One proposed mechanism linking meta-inflammation and chronic disease is the alteration of the gut microbiome. For example, changes in the gut microbiota have been linked to low back pain [24]. In a two-sample Mendelian randomized control study, it was found that lower levels of *Ruminococcaceae* and *Lactobacillaceae* in the gut flora were strong predictors of a higher risk of back pain [24].

Potential methods for addressing chronic pain through diet interventions targeting the microbiome have been explored in the management of chronic pain associated with endometriosis. Researchers found that low-FODMAP, Mediterranean, and gluten-free diets resulted in decreased reported pain [22]. Additionally, supplementation with Lactobacillus probiotics, omega-3 fatty acids, and vitamin D lowered pain. All of these interventions were thought to address microbiome alteration by increasing dietary fiber, increasing the consumption of foods that are more readily digested by existing gut flora, and supporting the growth of commensal bacteria in the gut [22].

More evidence supporting the importance of increased dietary fiber is the effect of short-chain fatty acids on microglia, neuroinflammation, and homeostatic signaling. The principal metabolites produced by microbiota through the anaerobic fermentation of indigestible polysaccharides, such as dietary fiber, are short-chain fatty acids [25]. It has been demonstrated that increased intake of dietary fiber reduces the risk of developing metabolic diseases, possibly due to changes in gut microbiome composition and diversity, with increased production of short-chain fatty acids [25]. Additionally, activation of neuronal receptors by butyrate and propionate (short-chain fatty acids) leads to suppression of the activity of appetite-stimulating orexigenic neurons in the hypothalamus, ultimately modulating signaling to the appetite-stimulating ghrelin receptor, thereby contributing to appetite control [25].

Proposed interventions for addressing metabolic and meta-inflammation

Dietary Modification

Diet-based interventions offer an alternative method for managing meta-inflammation, which may also reduce chronic pain through anti-inflammatory mechanisms. These dietary modifications provide a less invasive, more accessible, and holistic approach to addressing inflammation. Below are several dietary strategies that have been studied for their impact on inflammation and pain reduction.

Mediterranean diet (MD): The MD emphasizes fruits, vegetables, whole grains, nuts, seeds, and healthy fats, such as olive oil, along with moderate consumption of fish and poultry. Both observational and randomized controlled trials have demonstrated that the MD supports weight loss and improves cardiovascular surrogates, including waist-to-hip ratio, lipid profiles, and inflammatory markers [26]. Its anti-inflammatory benefits are attributed to several mechanisms, such as anti-inflammatory nutrients, gut microbiome modulation, and pain reduction.

For anti-inflammatory nutrients, the MD is rich in antioxidants, polyphenols, and omega-3 fatty acids, which reduce oxidative stress and suppress pro-inflammatory cytokines, such as IL-6, TNF-α, and CRP. Omega-3 fatty acids from fatty fish (e.g., salmon and mackerel) are particularly effective in lowering inflammatory markers involved in cardiovascular disease and chronic pain [27].

For gut microbiome modulation, the MD promotes a healthy gut microbiome, which is known to be closely linked to lower systemic inflammation. High dietary fiber from plant-based foods supports beneficial bacterial species that reduce endotoxemia - a key contributor to meta-inflammation. Specifically, the MD is associated with increased abundance of short-chain fatty acid-producing bacteria (e.g., *Clostridium leptum *and* Eubacterium rectale*), *Bifidobacteria*, *Bacteroides*, and *Faecalibacterium prausnitzii*, and decreased growth of *Firmicutes* and *Blautia *species [26]. These microbial shifts modulate inflammatory pathways via the NLRP3 inflammasome, toll-like receptor 4 (TLR4) signaling, and macrophage activity in adipose tissue [27].

For pain reduction, clinical studies suggest that adherence to the MD alleviates pain in inflammatory conditions, such as rheumatoid arthritis and fibromyalgia, by reducing systemic inflammation and enhancing metabolic function [27-30].

DASH (Dietary Approaches to Stop Hypertension) diet: The DASH diet was originally designed to reduce blood pressure but also confers anti-inflammatory effects. It emphasizes fruits, vegetables, whole grains, low-fat dairy, and lean proteins, such as fish and poultry, while limiting sodium, red meat, and added sugars. The DASH diet may reduce inflammation and chronic pain in the following ways: inflammation reduction, reduction in meta-inflammation, and improved endothelial function.

For inflammation reduction, in a meta-analysis comparing the inflammatory changes associated with varying levels of vegetables in diets, it was found that diets highest in legumes, leafy greens, and other whole vegetables had significantly lower CRP, fibrinogen, and leukocyte counts compared to diets high in animal-based products and red meats [31]. These inflammatory biomarker changes are attributed to vegetarian-based diets being high in flavonoids, carotenoids, and phytochemicals, which have direct antioxidant and anti-inflammatory properties [31]. 

For reduction in meta-inflammation, the DASH diet supports cardiovascular and metabolic health, thereby mitigating meta-inflammation [32]. Its emphasis on lean proteins and low sodium intake has been associated with reduced CRP and TNF-α levels. In a randomized clinical trial comparing the DASH diet to a calorie-deficit diet, the DASH group showed significant reductions in serum levels of hemoglobin A1c (HbA1c), TLR4, MCP-1, and lipopolysaccharides - even after adjusting for baseline values and weight change [32].

For improved endothelial function, the DASH diet’s beneficial effects on vascular function and blood pressure regulation may contribute to reduced inflammation and pain, particularly in patients with vascular-related pain syndromes [29,33].

MyPlate: MyPlate was created in 2011 to simplify dietary recommendations to incorporate increased intake of whole fruits, whole grains, and low-fat dairy products at each meal by creating a proportioned plate [34]. The MyPlate recommendations are associated with lowering levels of acute phase reactant CRP and HbA1c in diabetic patients [34]. The same benefits are likely possible for the reduction of meta-inflammation.

Specific nutrient recommendations: In addition to broad dietary patterns, several specific nutrients and dietary changes have been shown to help reduce pain through their effects on inflammation and metabolic health: omega-3 fatty acids, polyphenols, curcumin (from turmeric), vitamin D, and magnesium

Omega-3s, especially from fish oils, are known to reduce pro-inflammatory cytokines such as IL-1β, IL-6, and TNF-α, which play key roles in both meta-inflammation and pain perception [35]. Studies show that supplementing with omega-3s can reduce joint pain and improve functional outcomes in conditions such as arthritis [35]. However, excess consumption of omega-6 fatty acids leads to a pro-inflammatory state. It is therefore important to consider the ratio of omega-3 to omega-6, as elevated ratios are associated with complex pain syndromes [36]. Maintaining a lower, balanced omega-6/omega-3 fatty acid ratio is essential for reducing the risk of chronic diseases, managing inflammation, and potentially preventing obesity. 

Polyphenol-rich foods such as berries, dark chocolate, and green tea have been shown to modulate inflammatory pathways, including reducing nuclear factor kappa B (NF-κB) activation [37]. These bioactive compounds can decrease oxidative stress and cytokine production, contributing to the reduction of chronic inflammation and pain [37].

Curcumin is a potent anti-inflammatory agent that has been shown to inhibit NF-κB and cyclooxygenase-2 (COX-2), both involved in inflammatory pain pathways [38]. Supplementation with curcumin has been linked to reductions in joint pain and swelling, particularly in inflammatory conditions such as osteoarthritis and rheumatoid arthritis [38].

Low levels of vitamin D have been associated with inflammatory states. Vitamin D helps regulate the immune response and is involved in maintaining levels of pro-inflammatory cytokines. Supplementation in deficient individuals has been shown to improve symptoms of chronic musculoskeletal pain [39].

Magnesium plays a role in reducing inflammatory biomarkers, including CRP, and modulates pain perception by acting as a natural N-methyl-D-aspartate (NMDA) receptor antagonist [40]. Dietary sources of magnesium, such as leafy greens, nuts, and seeds, or direct supplementation, may help reduce chronic pain, particularly in conditions like fibromyalgia and migraine [40].

Lifestyle Modification

Poor sleep quality is associated with immune system disturbances, including significantly elevated levels of IL-6 and CRP [20,41]. Sleep restriction is associated with greater perceived pain, which could be implicated in the underlying immune-inflammatory dysfunction [20]. Interestingly, sleep quality has been found to be more impactful than sleep quantity on inflammatory markers. Therefore, it is recommended to get seven to eight hours of quality sleep rather than excessively long durations of low-quality sleep in order to reduce meta-inflammation [41]. Interventions to improve sleep quality include improving nighttime routines, such as decreasing exposure to blue light screens within two hours of sleep onset, following circadian sleep cycles aligned with daylight hours, and participating in relaxation techniques before sleep. 

Metabolic Medical Management

We have discussed the vicious cycle of meta-inflammation, metabolic dysregulation, and chronic disease. Therefore, given the direct role of inflammation in the development of chronic disease and chronic pain, anti-inflammatory medical strategies can be used to stop the cycle of meta-inflammation.

The active process of inflammation resolution is controlled by specialized pro-resolving mediators and receptors, whose activation has been confirmed to reduce inflammation, clear microbes, and alleviate pain [4]. One method for activating this resolutory process is direct pharmacological targeting. Pharmacological interventions targeting inflammatory cytokines have been shown to reduce levels of CRP and IL-6, leading to decreases in cardiovascular events [42]. Drugs like canakinumab, which target inflammatory pathways, could potentially be used in future clinical trials to assess their efficacy in reducing pain ratings and inflammatory markers in chronic pain patients [42].

**Figure 3:**
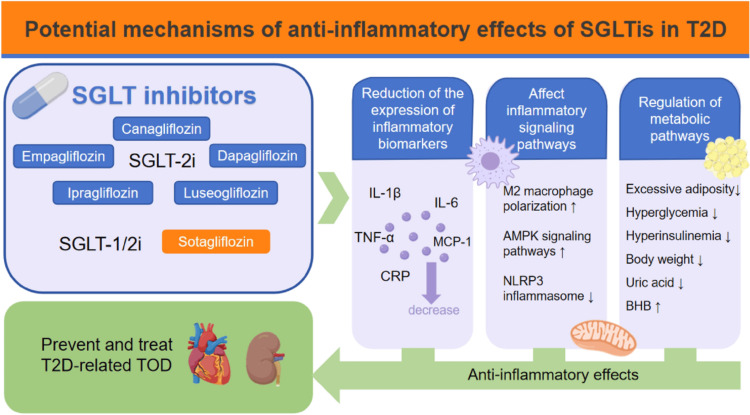
Anti-inflammatory mechanisms of SGLT2 inhibitors in the management of meta-inflammation This figure illustrates the role of sodium-glucose cotransporter-2 (SGLT2) inhibitors - specifically dapagliflozin and empagliflozin - in reducing meta-inflammation through the downregulation of inflammatory biomarkers. These hypoglycemic agents are shown to lower the expression of key cytokines, such as interleukin (IL)-1β and tumor necrosis factor-alpha (TNF-α), thereby mitigating chronic low-grade inflammation commonly associated with metabolic diseases. Their dual role in glycemic control and inflammation reduction supports their therapeutic potential in targeting meta-inflammation. Image credit: Reprinted from Zhang et al. [43]

Hypoglycemic agents, such as metformin, sodium-glucose cotransporter-2 (SGLT-2) inhibitors, and glucagon-like peptide-1 (GLP-1) agonists, not only have hypoglycemic effects to manage diabetes but additionally have anti-inflammatory properties, which likely contribute to the overall efficacy of these medications in controlling metabolic disease [30]. In fact, it was found in clinical trials that metformin is associated with decreased NF-κB, TNF-α, IL-1β, and IL-6 [30]. In further clinical trials, SGLT-2 inhibitors were associated with decreased IFN-λ, IL-6, TNF-α, and CRP [30]. Additional clinical trials showed an association between GLP-1 agonists and decreased IL-1β, IL-6, and TNF-α, and increased adiponectin [30]. Figure 3 summarizes the effects of metabolic medical management and lifestyle modifications on inflammatory markers in treating meta-inflammation.

Limitations

Research exists on the new concept of meta-inflammation, but research into its complete effects is limited - and even more limited when searching peer-reviewed medical databases. This review relied on articles citing the pathophysiology of meta-inflammation and its association with other chronic diseases to theoretically extrapolate a relationship with chronic pain.

Future recommendations

In order to confirm the relationship between meta-inflammation and pain, clinical trials should be conducted in which serum markers of inflammation are monitored in patients with chronic pain.

Incorporating dietary modification strategies into chronic pain management offers a non-invasive and more comprehensive approach to reducing meta-inflammation, ultimately improving pain outcomes. To accomplish the goal of providing adequate nutrition counseling to chronic pain patients, nutritionists educated on the topics of meta-inflammation control should be employed by the pain clinics. Alternatively, pain medicine specialists themselves could receive additional training in chronic inflammation - including nutrition, lifestyle modifications, and stress management - for the more complete management of patients with chronic pain and meta-inflammation.

Unfortunately, U.S. medical schools are only recommended to have 25 hours of coursework dedicated to nutrition. Even with this recommendation, the actual average number of nutrition education hours is only 19 [44]. Increasing the time dedicated to nutrition education during medical school would provide future physicians with the tools necessary to make these dietary recommendations in practice.

Outside of hiring a nutritionist for the pain clinics, the simple addition of implementing MyPlate, nutrient-guided additions, MD, or DASH diet recommendations to the general pain management patient instructions may be helpful. Along with these management instructions, routine testing for markers of inflammation can be done as a general data point for all pain specialists. A longitudinal study, after introducing these recommendations in chronic pain clinics, would be useful in evaluating this relationship.

## Conclusions

Chronic pain should be recognized as a distinct disease state, independent from other chronic conditions. Meta-inflammation plays a critical role in both chronic disease and pain, therefore necessitating its direct management in chronic pain treatment protocols. In addition to pharmacologic management, pain management practitioners may incorporate standardized dietary and lifestyle interventions aimed at reducing meta-inflammation to effectively treat chronic pain. Pharmacologic strategies targeting inflammatory pathways, like canakinumab and hypoglycemic agents (e.g., metformin, SGLT-2 inhibitors, and GLP-1 agonists), show a reduction in inflammatory markers such as IL-6, TNF-α, and CRP. Non-pharmacologic dietary approaches, such as the MD and DASH diets, have been found to lower systemic inflammation through the support of gut microbiota and direct intake of anti-inflammatory nutrients, which include omega-3 fatty acids, polyphenols, and fiber. Specific nutrients, such as vitamin D, magnesium, and curcumin, also play specific roles in modulating inflammation and pain by supporting immune health and reducing oxidative stress. Other lifestyle modifications, such as optimizing sleep quality for a duration of seven to eight hours daily, have demonstrated beneficial effects on reducing serum inflammatory markers. Integrating these lifestyle, dietary, and pharmacologic interventions offers a more comprehensive, non-invasive approach for managing chronic pain by reducing meta-inflammation and supporting metabolic health.
